# Concomitant optic nerve transection and chorioretinitis sclopetaria

**DOI:** 10.1186/1471-2415-5-29

**Published:** 2005-12-22

**Authors:** Mehrdad Mohammadpour, Masoud Soheilian

**Affiliations:** 1 Cornea fellow, Ophthalmic Research Center, Labbafinejad Medical Center-9^th ^Boostan, Pasdarn Street Tehran- Iran; 2 Cheif Professor of Ophthalmology, Ophthalmic Research Center, Labbafinejad Medical Center-9^th ^Boostan, Pasdarn Street Tehran- Iran

## Abstract

**Background:**

Optic nerve transection and chorioretinitis sclopetaria may occur following blunt ocular trauma. However, simultaneous occurrence has not yet been reported. We report the first case of concomitant optic nerve transection and chorioretinitis sclopetaria.

**Case presentation:**

A 12- year- old boy with history of BB gun injury to his right eye was referred for loss of vision. His visual acuity was counting fingers at one meter in the right eye and with 3+ relative afferent pupillary defect (RAPD).

On slit lamp examination, the right eye appeared normal except for 1+ vitreous reaction. Fundus examination of the right eye revealed a pale disc with superior retinal scar and diffuse submacular fibrosis compatible with chorioretinitis sclopetaria. Orbital CT- scans showed transection of the optic nerve by the BB gun pellet, which was lodged at the orbital apex.

**Conclusion:**

BB gun injury may cause concomitant optic nerve transection and chorioretinitissclopetaria.

## Background

Blunt nonpenetrating post-traumatic maculopathies have diverse manifestations including choroidal rupture, posttraumatic macular hole, commotio retinae (Berlin's edema), Purtscher retinopathy and chorioretinitis sclopetaria [1]. Chorioretinitis sclopetaria is the result of traumatic chorioretinal rupture followed by marked fibrovascular proliferation with variable replacement of the choroid and retina with no retinal detachment [1].

Contusion force may lead to choroidal ruptures with hyperplasia and migration of the retinal pigment epithelium into the retina and choroid, epiretinal membrane formation, loss of photoreceptors and marked hemiatrophy of the optic nerve [2].

Optic nerve injury may cause traumatic optic neuropathy and optic nerve transection [3]. There is no definite treatment for optic nerve transection or sclopetaria but recent investigations show successful intravitreal transplants of Schwann cells and fibroblasts in axotomized retinal ganglion cells in animal models [4]. Metallic orbital foreign bodies such as BB gun pellets may cause all the above-mentioned injuries, however concomitant optic nerve transection and chorioretinitis sclopetaria in an intact globe has not yet been reported.

## Case presentation

**Figure 1:**
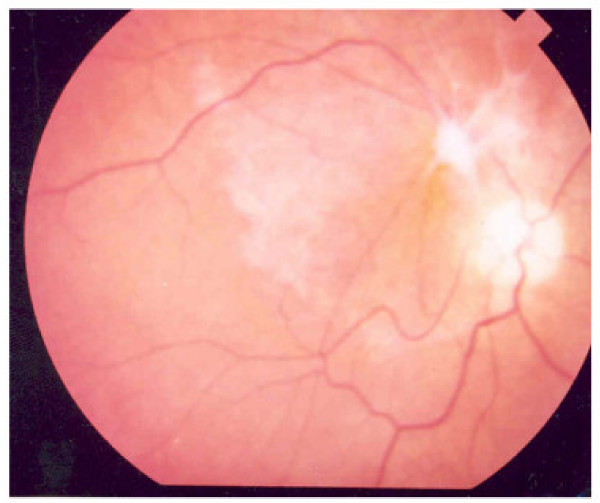
Colour fundus photograph of the right eye showing macular scar and massive subretinal fibrosis and a pale optic nerve.

**Figure 2:**
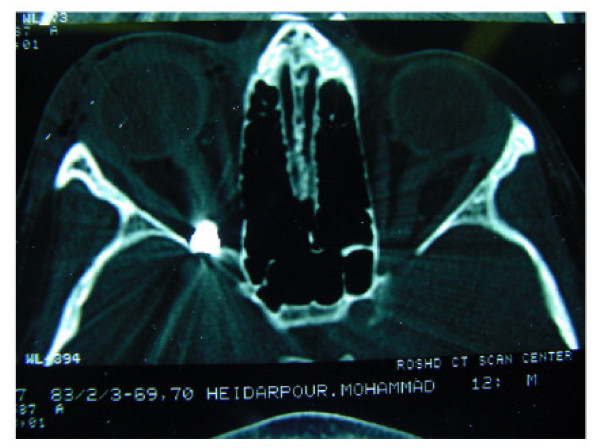
Axial orbital CT scan showing optic nerve transection at right orbital apex.

A 12- year- old boy with history of BB gun entrance to his right orbital space in May 2004 referred to us in September 2004 with loss of visual acuity, that was counting fingers at one meter. The relative afferent pupillary defect (RAPD) was positive in the right eye. Slit lamp examinations of the right eye were unremarkable, except 1+ vitreous reaction. Intraocular pressure in the right eye was 10 mm-Hg without any medication. Fundus examinations of right eye showed a pale disc and a superior retinal scar and diffuse submacular fibrosis compatible with chorioretinitis sclopetaria (figure 1). Examinations of the left eye were unremarkable. Orbital CT- scans showed transection of the optic nerve by the BB gun pellet at the orbital apex (figure 2). Choroidal circulation was markedly impaired in earlier fluorescein angiography (FAG) reports. However, due to the low quality of the images taken at another center during the acute phase of disease with intense vitreous reaction of the right eye, the images are not shown.

Due to end stage optic atrophy, diffuse submacular fibrosis and inert nature of the metallic foreign body in the right orbital space no surgical intervention was planned for the patient.

## Conclusion

Retained intraorbital metallic foreign bodies may accompany chorioretinitis scleopetaria, commotio retinae, vitreous hemorrhages or may be innocent [5]. In the case of BB gun pellets, the usual management is conservative due to the inert nature of this type of metallic foreign bodies [5]. There are considerable reported cases with chorioretinitis sclopetaria due to BB gun injuries [3]. The characteristic pattern of choroidal and retinal changes caused by a high velocity projectile object passing through the orbit, in close proximity to the globe is usually seen in this condition.

The optic nerve may be transected by direct or indirect trauma and results in permanent visual loss. The diagnosis of traumatic optic neuropathy is not always straightforward. The diagnosis should be established only based on clear objective findings, a relative afferent pupillary defect or a pathological flash – evoked visual response [3]. The most important factor in determining the force of injury is the velocity. The higher the velocity of the projectile object, the greater the tissue disruption at impact due to kinetic energy. It takes an impact velocity of 132 fps to penetrate the human eye, 290 fps to penetrate skin, and 331 fps for complete passage through the skin and into soft tissues. The shock wave energy released by the projectile object is considered as the main offender causing choroidal and subsequently retinal injury [6]. The presumed track of BB in this case is through the right upper eyelid, adjacent to the posterior sclera into the muscle cone and finally transecting the optic nerve and lodging in the orbital space.

Treatment of traumatic optic neuropathy consists of surgical decompression of optic nerve (even without direct injury to the nerve), use of megadose steroids or both in combination, even though, spontaneous improvement may rarely occur. In the case of complete optic nerve transection there is no definite treatment and it generally leads to optic nerve atrophy and permanent visual loss. [3] Recently, there is one report of intravitreal transplants of Schwann cells and fibroblasts in axotomized retinal ganglion cells in rats [5], but there is no report of successful treatment of optic nerve transection in humans.

To the best of our knowledge, this patient is the first reported case of concomitant optic nerve transection and chorioretinitis sclopetaria due to gunshot injury.

## Competing interests

The author(s) declare that they have no competing interests.

## Contribution of the authors

***Mehrdad Mohammadpour***, *MD, Cornea fellow, Ophthalmic Research Center is the corresponding author and *Masoud Soheilian, *MD, Full Professor of Ophthalmology, Labbafinrjad Medical Center is the retina consultant*.

## Pre-publication history

The pre-publication history for this paper can be accessed here:
